# Left Ventricular Noncompaction Is More Prevalent in Ventricular Septal Defect Than Other Congenital Heart Defects: A Morphological Study

**DOI:** 10.3390/jcdd7040039

**Published:** 2020-09-25

**Authors:** Laís Costa Marques, Gabriel Romero Liguori, Ana Carolina Amarante Souza, Vera Demarchi Aiello

**Affiliations:** 1 Laboratory of Pathology, Heart Institute (InCor), Hospital das Clinicas HCFMUSP, Faculdade de Medicina, Universidade de Sao Paulo, Sao Paulo 05403-000, Brazil; laiscostamarques@gmail.com (L.C.M.); gabriel.liguori@usp.br (G.R.L.); anacarol.amarante@gmail.com (A.C.A.S.); 2 Laboratory for Tissue Engineering and Organ Fabrication (LTEOF), Heart Institute (InCor), Hospital das Clinicas HCFMUSP, Faculdade de Medicina, Universidade de Sao Paulo, Sao Paulo 05403-000, Brazil

**Keywords:** congenital heart defects, left ventricular noncompaction, ventricular septal defects

## Abstract

Left ventricular noncompaction (LVNC) is a condition characterized by prominent ventricular trabeculae and deep intertrabecular recesses and has been described as a possible substrate for arrhythmias, thromboembolism, and heart failure. Herein, we explored the prevalence of LVNC morphology among hearts with congenital heart defects (CHD). We examined 259 postnatal hearts with one of the following CHD: isolated ventricular septal defect (VSD); isolated atrial septal defect (ASD); atrioventricular septal defect (AVSD); transposition of the great arteries (TGA); isomerism of the atrial appendages (ISOM); Ebstein’s malformation (EB); Tetralogy of Fallot (TF). Eleven hearts from children who died of non-cardiovascular causes were used as controls. The thickness of the compacted and non-compacted left ventricular myocardial wall was determined and the specimens classified as presenting or not LVNC morphology according to three criteria, as proposed by Chin, Jenni, and Petersen. Normal hearts did not present LVNC, but the CHD group presented different percentages of LVNC in at least one diagnostic criterium. The prevalence of LVNC was respectively, according to Chin’s, Jenni´s and Petersen´s methods: for VSD—54.2%, 35.4%, and 12.5%; ASD—8.3%, 8.3%, and 8.3%; AVSD—2.9%, 2.9%, and 0.0%; TGA—22.6%, 17%, and 5.7%; ISOM—7.1%, 7.1%, and 7.1%; EB—28.6%, 9.5%, and 0.0%; TF—5.9%. 2.9%, and 2.9%. VSD hearts showed a significantly greater risk of presenting LVNC when compared to controls (Chin and Jenni criteria). No other CHD presented similar risk. Current results show some agreement with previous studies, such as LVNC morphology being more prevalent in VSDs. Nonetheless, this is a morphological study and cannot be correlated with symptoms or severity of the CHD.

## 1. Introduction

Left ventricular noncompaction (LVNC) is a condition first described in the late 1980s [1]. It is defined as a heart morphologically characterized by excessively prominent ventricular trabeculations and deep intertrabecular recesses that communicate with the ventricular cavity [2]. Diagnostic criteria vary according to different authors [3]. There are two-dimensional echocardiography criteria, such as those described by Chin [4], Jenni [5], and Stöllberger [6], as well as criteria based on cardiac magnetic resonance, as proposed by Petersen [7] and Jacquier [8]. Usually, echocardiography is the first line diagnostic modality [3]. 

During the last 30 years, LVNC has gained increasing recognition and a great number of authors focused on investigating and describing this anatomic abnormality and its relationship with clinical and pathological findings. Some reports associated the presence of LVNC with the occurrence of heart failure, malignant arrhythmias, and thromboembolic events [9,10,11]. These symptoms became considered as the tripod of LVNC [4,12]. In children with congenital heart disease (CHD) undergoing heart surgery, LVNC is known to be associated with longer hospital length of stay and higher perioperative complications compared to CHD-only patients without myocardial abnormalities [13].

One of the relevant discussions regarding LVNC is how the disease develops. While some authors believe LVNC is a failure of compaction of the preexisting trabecular formation of the heart [14], others say that the compacted component of the heart is well formed and the excessive trabeculations are a proliferation defect [15]. Although the most commonly accepted hypothesis states that the compacted component of the heart derives from initial trabeculations undergoing compaction, Anderson et al. showed that, in the early stages of ventricular formation (Carnegie stages 15 and 16), a well formed compacted ventricle is already present, despite the existence of a thick layer of persisting trabecular myocardium [15]. 

**Figure 1:**
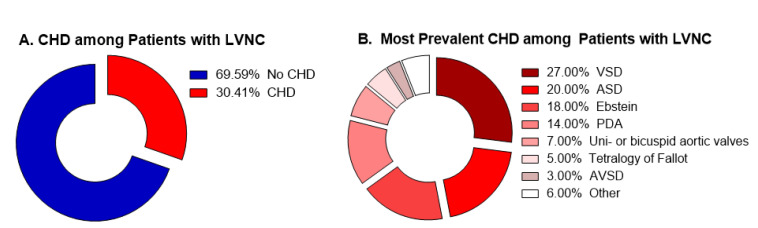
Summary of the current studies available in literature. (**A**) Percentage of patients with CHD among all the patients presenting LVNC. (**B**) Percentage of each CHD among all the patients presenting concomitant CHD/LVNC.

Given that congenital heart defects (CHD) are also established during heart development, there might be possible associations between CHD and LVNC derived from common genetic abnormalities. Understanding this association could guide both clinical practice, as well as anatomy, embryology and genetic studies aiming to understand the development of LVNC. Although many authors reported cases of patients presenting concomitant CHD and LVNC [16,17,18,19,20,21,22,23,24], to date, only four studies sought to establish epidemiological associations between the two entities. All of them, however, selected first patients known to have LVNC and looked for the prevalence of CHD among them. The data from these studies are summarized in Table 1 and Figure 1. 

Herein, we hypothesize that the presence of CHD is a major finding and the concomitant occurrence of LVNC is a complicator, not the opposite. Thus, we investigated the prevalence of LVNC in heart specimens of patients with CHD.

## 2. Materials and Methods 

From the anatomical archives of the Laboratory of Pathology of the Heart Institute (InCor), University of Sao Paulo Medical School, Brazil, we identified 279 postnatal hearts with one of the following congenital heart defects: (1) isolated ventricular septal defect (VSD); (2) isolated atrial septal defect (ASD); (3) atrioventricular septal defect (AVSD); (4) transposition of the great arteries (TGA); (5) isomerism of the atrial appendages; (6) Ebstein anomaly; and (7) tetralogy of Fallot. In 20 hearts, the analysis was not possible, either because of previous dissection or previous surgery; the remaining 259 hearts were examined.

The myocardial thickness of the compacted and non-compacted regions in each heart were determined for the left ventricular inlet, apex and outlet muscular walls. Measurements were performed in the long axis. The points of measurements were: at the inlet wall, in a point immediately proximal to the insertion of the anterolateral papillary muscle of the left ventricle; at the apical wall; at the left ventricular free wall, along the surface of myocardial section, at the distal third of the length between the apex and the line of aortic valve insertion, considered as the measurement of the ventricular outlet. 

According to the 17-segmental model as described by Partridge and Anderson [29], the inlet measurement was taken perpendicular to the compacted myocardium at segment #7, with the anatomical section corresponding to the vertical long axis image; the apical measurement at segment #17 of the same axis and the outlet measurement at the median point of segment #8 of the echocardiographic parasternal long axis view. The papillary muscles were not included in the measurements.

According to the values found for the apex, specimens were classified as presenting or not LVNC according to three categorization criteria, as proposed by Chin, Jenni, and Petersen (Table 2).

This study was conducted in accordance with the Declaration of Helsinki, and the protocol was approved by the “Comissão de Ética para análise de projetos de pesquisa, Hospital das Clínicas da Faculdade de Medicina da Universidade de São Paulo”, protocol #534.621 (19/02/2014). 

### Statistical Analysis

Data were expressed as the median and interquartile ranges (IQR). Comparisons for the quantitative variables were made through Kruskal–Wallis one-way analysis of variance with post-hoc Holm–Sidak test, or through Mann–Whitney test. To assess qualitative associations, Chi-square or Fisher’s exact test were conducted and odds ratio (OR) was determined. All statistical analyses were performed using GraphPad Prism 6.0 (Graphpad Software Inc., La Jolla, CA, USA).

## 3. Results

### 3.1. Sample Characteristics

A total of 259 patients were included in the present study. Briefly, 87.3% of the patients were children, i.e., age below 18-year-old and 51.0% were female. Fifty-one (19.7%) patients presented ventricular septal defect (VSD), 16 (6.2%) atrial septal defect, 41 (15.8%) atrioventricular septal defect, 54 (20.8%) transposition of the great arteries, 29 (11.2%) atrial isomerism, 23 (8.9%) Ebstein´s anomaly, and 34 (13.1%) tetralogy of Fallot. Regarding the VSD types, 74.5% were isolated perimembranous, 5.8% were isolated muscular, 11.7% were subarterial, and in 7.8% there was more than one defect (two cases with more than one muscular defect and two with one perimembranous and one muscular). The atrioventricular septal defects were divided in: complete form (one orifice, interatrial and interventricular communications)—72%; partial form (two orifices, communication at the atrial level only)—28%. Cases of transposition of the great arteries (TGA) comprised: intact ventricular septum, interatrial communication only—52.2%; interatrial communication associated with subpulmonary stenosis—2.1%; ventricular septal defect—45.6%. Hearts with isomerism of the atrial appendages had right isomerism—54.2% and left isomerism—45.8%. 

Eleven (4.9%) patients without congenital heart disease were included as healthy controls. The overview of the demographic data stratified for type of congenital heart defect is described in Table 3.

### 3.2. Both Short and Long Axis of the Heart Can Be Used for Noncompaction Measurements

**Figure 2:**
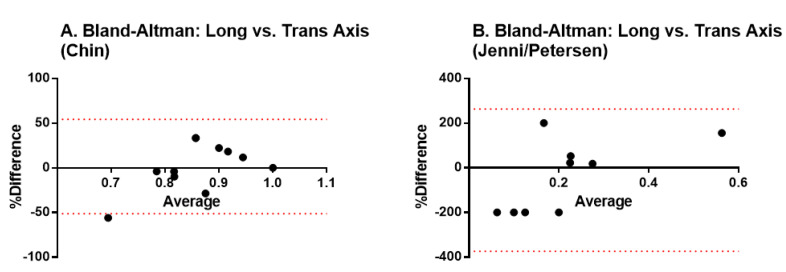
Bland-Altman tests to validate the agreement between measurements performed in the short versus the long axis of the heart. (**A**) transversal axis; (**B**) longitudinal axis

We first analyzed how the acquisition method influences the diagnostic results. We used the Bland-Altman test for analyzing the agreement between the measurements performed in the short versus the long axis of the heart. Results indicate there is no fixed bias resulting in statistically significant differences among the methods, thus, both may be used interchangeably (Figure 2). 

### 3.3. Different Ventricular Walls Present Different Degrees of LVNC

Ventricular trabeculation was evaluated in three left ventricle portions: the inlet, apex, and outlet. Then, the Chin, Jenni, and Petersen analysis were performed for each of these portions. 

**Figure 3:**
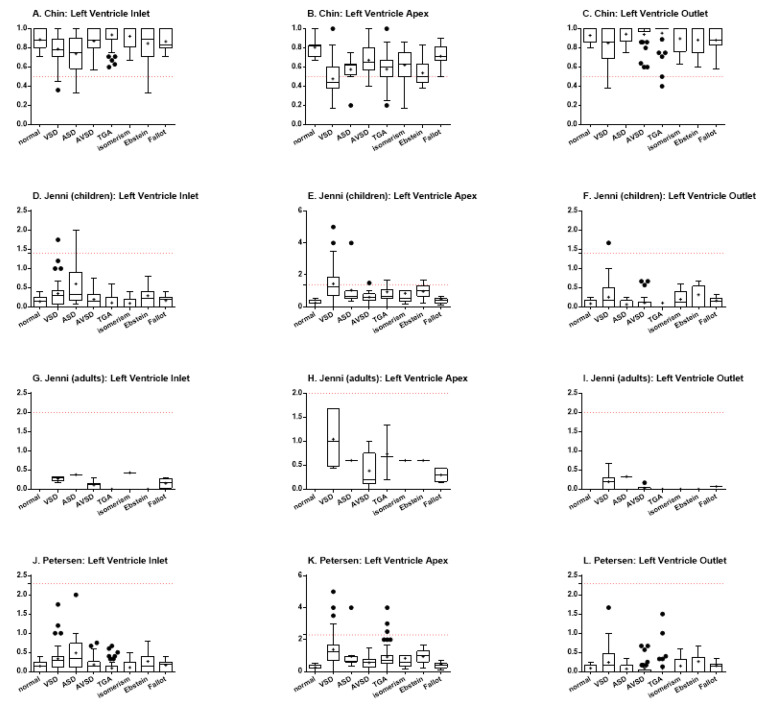
Indexes of ventricular trabeculation, as evaluated by Chin, Jenni, and Petersen methods, in the inlet, apex and outlet muscular walls of the left ventricle. The red dotted lines represent the threshold for LVNC for each criterium.(**A**) Chin, left ventricle inlet; (**B**) Chin, left ventricle apex; (**C**) Chin, left ventricle outlet; (**D**) Jenni, (children) left ventricle inlet; (**E**) Jenni, (children) left ventricle apex; (**F**) Jenni, (children) left ventricle outlet; (**G**) Jenni, (adults) left ventricle inlet; (**H**) Jenni, (adults) left ventricle apex; (**I**) Jenni, (adults) left ventricle outlet; (**J**) Petersen, left ventricle inlet; (**K**) Petersen, left ventricle apex; (**L**) Petersen, left ventricle outlet.

Using Chin’s method, it was possible to observe statistically significant differences among the specimens with VSD and TGA (*p* < 0.0001), VSD and isomerism (*p* = 0.0031), ASD and TGA (*p* = 0.0031), and ASD and isomerism (*p* = 0.0397) for the left ventricle inlet (Figure 3A). When analyzing the ventricular apex, there were differences among normal heart and hearts with VSD (*p* < 0.0001), TGA (*p* = 0.0011), and Ebstein (*p* = 0.0002) (Figure 3B). There were also differences among hearts with VSD and AVSD (*p* < 0.0001), isomerism (*p* = 0.0021), and Fallot (*p* < 0.0001), besides TGA vs. Fallot (*p* = 0.0016) and Ebstein vs. Fallot (*p* = 0.0005). Finally, the analysis of the left ventricle outlet showed statistically significant differences when comparing hearts with VSD and TGA (*p* = 0.0034) and TGA vs. Fallot (*p* = 0.0059) (Figure 3C). For all the three portions, the hearts with ventricular septal defect demonstrated an increased proportion of trabeculated ventricular wall (focal noncompaction), when compared to the normal heart or to other congenital heart defects. 

Jenni’s method includes the stratification of patients between children and adults. Thus, due to the limited number of adult patients, the data for this group was plotted (Figure 3G–I), but could not be statistically analyzed. When analyzing the data for the pediatric group, however, some differences could also be found. In regard to the left ventricle inlet, differences were evidenced between hearts with VSD and TGA (*p* = 0.0004) and VSD and isomerism (*p* = 0.0019) (Figure 3D). The same was found when comparing hearts with ASD and hearts with TGA (*p* = 0.0081) or isomerism (*p* = 0.0096). When analyzing the heart apex, the normal hearts demonstrated less trabeculation than hearts with VSD (*p* < 0.0001), TGA (*p* = 0.0024), and Ebstein (*p* = 0.0002), while hearts with VSD showed more trabeculation than those with AVSD (*p* = 0.0015), isomerism (*p* = 0.0019), and Fallot (*p* < 0.0001) (Figure 3E). It was also possible to describe differences among hearts with TGA vs. Fallot (*p* = 0.0241) and Ebstein vs. Fallot (*p* = 0.0019). For the left ventricular outlet, there was only difference between hearts with VSD and TGA (*p* = 0.0390), so that the former were more trabeculated than the later (Figure 3F). 

Petersen’s method, in turn, although performing data acquisition differently than Jenni’s method, uses the same calculation to define trabeculation. However, this method does not separate children and adults, so that the data from all the patients could be analyzed together. The findings showed differences between hearts with VSD and those with TGA (*p* < 0.0001) and isomerism (*p* = 0.0031), besides also between hearts with ASD and TGA (*p* = 0.0031) and isomerism (*p* = 0.0399), for the left ventricle inlet (Figure 3J). The analysis of the apex portion demonstrated statistically significant differences between the normal hearts and hearts with VSD (*p* < 0.0001), ASD (*p* = 0.0360), TGA (*p* = 0.0012), and Ebstein (*p* = 0.0002) (Figure 3K). There were also differences between the hearts with VSD and AVSD (*p* < 0.0001), isomerism (*p* = 0.0050), and Fallot (*p* < 0.0001). Differences among hearts with TGA and Fallot (*p* = 0.0016), besides Ebstein and Fallot (*p* = 0.0005), could also be described. Finally, for the left ventricle outlet, Petersen’s analysis demonstrated differences between the hearts with VSD vs. TGA (*p* = 0.0034) and TGA vs. Fallot (*p* = 0.0059) (Figure 3L).

**Figure 4:**
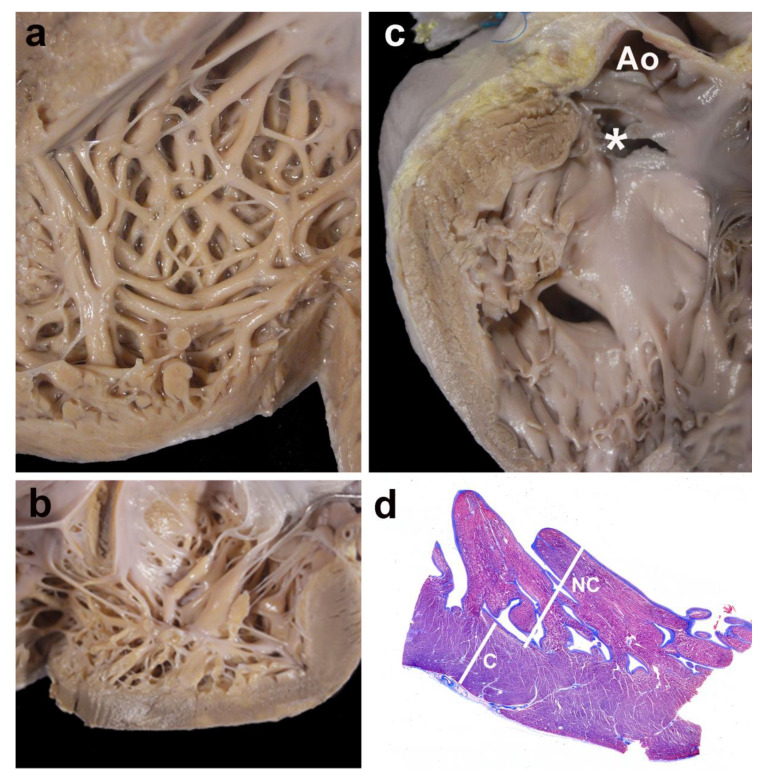
Gross and histological morphology. Four examples of cases showing focal non-compaction of the left ventricle. (**a**) and (**b**) Left ventricular wall of two cases with Ebstein’s anomaly of the tricuspid valve. The apical regions show a thick noncompacted layer when compared to the compacted myocardium. In (**c**), a case with a muscular trabecular ventricular septal defect shows a focal area of noncompaction at the left ventricular outlet free wall (* the membranous septum has been accidentally torn, but was originally intact); Ao—opened aorta. Panel (**d**) depicts the histological aspect of the apical region of a case with complete transposition of the great arteries, although histology was not used for measurements. The noncompacted layer (NC) is thicker than the compacted myocardium (C); Masson´s trichrome stain.

Figure 4 shows four examples of cases with LVNC. 

### 3.4. LVNC Is More Often Associated with Ventricular Septal Defect than Other Congenital Heart Diseases

The odds ratio for presenting left ventricular noncompaction (hyper-trabeculation) was calculated relative to the normal hearts. The findings were summarized in Table 4. Briefly, hearts presenting VSD showed a significantly increase in the risk of presenting LVNC when compared to normal hearts when using the Chin (*p* = 0.0013; OR 27.09; 95CI 1.51–486.10) and Jenni (*p* = 0.0241; OR 12.78; 95CI 0.71–230.3) criteria. As also demonstrated by the quantitative analysis (Figure 3), the Petersen method was the most conservative and did not evidence any differences among the groups. 

**Figure 5:**
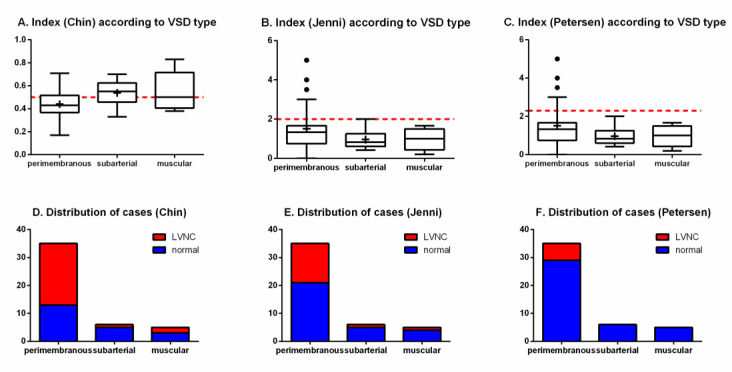
Indexes of left ventricular trabeculation according to VSD type (superior panels) and distribution of cases (inferior panels), as evaluated by Chin, Jenni, and Petersen methods, in. The red dotted lines represent the threshold for LVNC for each criterium. (**A**) index (hin) according to VSD type; (**B**) index (Jenni) accrding to VSD type; (**C**) index (Petersen) according to VSD type; (**D**) distribution of cases (Chin); (**E**) distribution of cases (Jenni); (**F**) distribution of cases (Petersen).

When considering the types of VSDs, no significant difference was detected both in the quantitative indexes (Chin, *p* = 0.1680; Jenni/Petersen, *p* = 0.2357) and in the proportion of cases of LVNC (Chin, *p* = 0.0876; Jenni, *p* = 0.4128; Petersen, *p* = 0.3361) (Figure 5).

Two of the four specimens with more than one VSD (2 with 1 perimembranous + 1 muscular and 2 with more than one muscular) were positive for the LVNC phenotype at the apical measurement, but the statistical comparison did not find differences in prevalence in this group, possibly due to the small number of cases.

Hearts with TGA and Ebstein, although not reaching statistical significance, showed a trend to an increased risk of LVNC (TGA: *p* = 0.1064; OR 6.93; 95CI 0.38–126.1; Ebstein: *p* = 0.0711; OR 27.09; 95CI 0.49–189.2) when analyzed by Chin’s method. 

### 3.5. Different LVNC Diagnostic Criteria Lead to Important Differences in Outcomes

When the prevalence of LVNC was ranked for the congenital heart diseases according to the three different diagnostic methods, differences could be evidenced among them (Table 5). Still, for all the methods, hearts with VSD were those presenting the higher prevalence of LVNC. Oppositely, also for all the methods, hearts with AVSD were those with the lower prevalence of LVNC. For the other diseases, there were differences among the methods.

For the hearts with TGA and atrial isomerism, samples were stratified according to the associated defects. Hearts with TGA were stratified among isolated TGA, TGA with ASD, TGA with ASD plus VSD, and TGA with VSD (Table 6). Hearts with atrial isomerism were stratified among isolated isomerism, isomerism with ASD, isomerism with ASD and VSD, and isomerism with AVSD, and other combinations (Table 7). All combinations were compared to hearts presenting the isolated defect, either TGA or isomerism. Although hearts with TGA and isomerism combined with ASD plus VSD showed the higher prevalence of LVNC for all the three diagnostic criteria, the only statistically significant difference was found for hearts with TGA combined with ASD plus VSD when using Jenni’s method (*p* = 0.0324; OR 10.67; 95CI 1.30–86.98). 

### 3.6. Prevalence of LVCN According to Age

The prevalence of LVNC was stratified for each disease between children and adults, and reported for the three diagnostic criteria. The summary of the findings is described in Table 8. In general, the prevalence of the disease was higher in children than in adults. 

### 3.7. Sex Influence on the Prevalence of LVCN

We investigated the influence of sex in the prevalence of LVNC. For this purpose, data were stratified, for each congenital heart disease and diagnostic method, between males and females. The results are summarized in Table 9. Briefly, the prevalence of LVNC in males and females varied according to the disease and was increased to either strata depending on the disease. The statistical analysis, however, could only evidence an increased risk for females to present LVNC among patients with VSD, when using the Petersen criteria (*p* = 0.0192; OR 15.40; 95CI 0.80–297.2). In these patients, while 22.7% of the female presented LVNC, no male patient had the disease. For all the other congenital heart defects and diagnostic criteria, no statistically significant differences could be found.

## 4. Discussion

Herein, we aimed to describe the prevalence of the noncompaction ventricular pattern among hearts with different types of congenital heart disease. We examined 259 specimens with seven different congenital heart defects (VSD, ASD, AVSD, TGA, isomerism, Ebstein malformation and tetralogy of Fallot) according to three classification criteria (Jenni, Chin, and Petersen). To the best of our knowledge, this is the largest sample describing associations between LVNC and CHD. We found that (1) the prevalence of LVNC varies when using different diagnostic criteria; and (2) VSD was the CHD with greater prevalence of LVNC for all the three criteria, followed by Ebstein and TGA—when using Chin or Jenni criteria—or ASD and isomerism—when using Petersen criteria. 

Many authors reported cases of patients presenting concomitant CHD and LVNC, including Ebstein [16,18,20,22], congenital atresia of the left main coronary artery [19], VSD [24], double-chambered left ventricle [23], patent ductus arteriosus [21], and also a complex congenital defect formed by *cor-triatriatum*, VSD, persistent left superior caval vein and an anomalous extracardiac vessel [17]. To date, however, only four studies sought to establish epidemiological associations between the two entities. In these studies, authors selected first patients known to have LVNC and looked for the prevalence of CHD among them. Taken together, they showed that, among patients with LVNC, 30.4% presented a CHD (range 19.1–78.0%). Of these patients, most of them had VSD, followed by ASD, Ebstein, PDA, uni- or bicuspid aortic valve, tetralogy of Fallot, and AVSD, respectively. While the tree studies using Jenni criteria found CHD prevalence between 19.1% and 29.5%, one study using Chin criteria found 78.0% prevalence of CHD among patients with LVNC. 

The prevalence of each CHD found by these four studies matches with the expected prevalence for CHD in the general population. Literature describes the most prevalent CHD as being VSD, ASD, PDA, pulmonary stenosis, and TOF [30]. Among these diseases, only pulmonary stenosis was not described as a major CHD among patients with LVNC. Interestingly, however, authors showed Ebstein malformation as one of the major CHD among patients with LVNC. This is in accordance with our findings, in which we demonstrated a high prevalence of LVNC among Ebstein patients. Thus, even with the disease not being a prevalent CHD, its prevalence among LVNC specimens is high. However, the same was not true for TGA, which was also shown to have a high prevalence of LVNC in our sample, but did not stand out in the other studies. 

An important limitation, both in our study as in the rest of literature, is the classification criteria for LVNC. As previously mentioned, several of them exist, Chin, Jenni, and Petersen being the most relevant of them. Each of these criteria uses different methodologies, not only the ratio threshold, but also the reference axis (short vs. long), the moment of acquisition (end-systolic vs. end-diastolic), and also the imaging technique (echocardiography vs. MRI). In the present study, we performed the gross analysis of heart specimens, thus, neither an echocardiography nor MRI. We analyzed the specimens sectioned in the long axis (as in Petersen) because they were already dissected on this axis, as this is the standard for necropsy studies in our institution. In order to analyze if the chosen axis would result in diagnostic bias, we performed the Bland-Altman test, which showed that there was no difference between the short and long-axis measurements; thus, both could be used interchangeably. Still, the classification criteria seem to be a major issue when stratifying patients with or without LVNC. While some criteria, such as Chin, are very permissive, resulting in high sensitivity and low specificity, others, like Petersen, are more restrictive, resulting in high specificity. In their original study, Petersen et al. [7] claim an 86% sensitivity and a 99% specificity, pointing out also that the sensitivity of cardiovascular magnetic resonance is increased as compared to the other methods of cardiac imaging. 

Another limitation of the study involving heart specimens is the lack of information about the contraction status of the myocardium. We admit that this uncertainty could be a bias in our study, but believe that it would not be important in determining the ratio between compacted and non-compacted layers, because contraction status would apply to both layers. 

Unfortunately, because we used necropsy hearts, we could not perform hemodynamic studies—as measuring ejection fraction, shortening fraction, etc.—and thus, we could not correlate our findings with the clinical status of the patient. Furthermore, in the majority of our cases such data were not available in the electronic health record. Several authors argue that hypertrabeculation of the left ventricle does not mean debilitated heart [15,31]. They defend that excessive trabeculation alone is not clinically relevant and there must be other modifications, such as reduced ejection fraction, fibrosis, and dilated left ventricle, in order to consider LVNC as a disease. These parameters, however, are already known hallmarks of cardiomyopathy. Thus, it is still difficult to define patients affected by LVNC, either by genetic or imaging diagnostic tools, with absolute certainty. Although for the general population the LVNC diagnosis might not necessarily lead to poor prognosis, this might not be true for children with CHD, which already have a diseased heart and, thus, may be more susceptible to the detrimental effect of hypertrabeculation. Hence, particularly in the case of patients with VSD (and maybe also Ebstein and TGA), diseases which we demonstrated to be a risk factor for LVNC, the screening could be beneficial for the management of the disease and possibly a feature to affect late follow-up after surgery. The recent description of LVNC as a trait in the MOGE(S) nosology system allows to recognize that the LVNC finding may possibly contribute to left ventricular dysfunction in coexisting morphofunctional disorders, such as the congenital heart defects [32]. 

Another point deserving discussion is that the diagnosis of LVNC was made in our series whenever a single region of the left ventricular wall was positive. Therefore, it would be better to describe “focal LVNC”, while in cases with dilated cardiomyopathy phenotype of the disease, the non-compacted pattern is usually diffuse. 

## 5. Conclusions

The prevalence of focal LVNC phenotype among CHD patients varies widely according to the criteria being used. In the present study, we showed that the Chin criteria are more permissive, while Petersen’s are more restrictive. According to both Chin and Jenni classification methods, VSD is a risk factor for LVNC. This is not true when using Petersen classification but, still, VSD was also the CHD with higher LVNC prevalence when using this classification method. Ebstein and TGA demonstrated trends towards representing a risk factor for LVNC, but could not be confirmed in the present study. Follow-up studies should be performed in large cohorts of living patients, children and adults, and include clinical data such as symptoms and exam results. 

## Figures and Tables

**Table 1:** Summary of the current studies available in literature about prevalence of congenial heart defects among cases of noncompaction.

Reference	Sample Size	Criteria	Total of CHD	Congenital Heart Defect (*n*)
Stähli (2013) [25]	202	Jenni	40 (19.8%)	Uni-or bicuspid aortic valves (7) Ebstein Anomaly (6) Tetralogy of Fallot (3) Aortic Coarctation (2)
Zuckerman (2010) [26]	50	Jenni	13 (26.0%)	VSD (7) ASD (4) PAPVC (1) Pulmonary atresia with intact ventricular septum (1)
Punn (2010) [27]	44	Jenni	13 (29.5%)	Ebstein Anomaly (7) AVSD (3) VSD (3) Tetralogy of Fallot (2) Pulmonary valve stenosis (2)
Tsai (2009) [28]	46	Chin	38 (82.6%)	VSD (17) ASD (16) PDA (14) Ebstein Anomaly (5)

CHD, congenital heart disease; VSD, ventricular septal defect; ASD, atrial septal defect; PAPVC, partial anomalous pulmonary venous connection; AVSD, atrioventricular septal defect; PDA, patent ductus arteriosus.

**Table 2:** Criteria for detection of LVNC in the clinical practice.

Criterium	Method	Acquisition	Moment	LVNC Threshold
Chin et al.	Echocardiography	Short axis	End-diastolic	C/T < 0.5
Jenni et al.	Echocardiography	Short axis	End-systolic	NC/C > 1.4 (or >2.0 in adults)
Petersen et al.	CMR	Long axis	End-diastolic	NC/C > 2.3

C: compacted; NC: non-compacted; T: total (compacted + non-compacted); CMR: cardiovascular magnetic resonance.

**Table 3:** Sample Characteristics.

CHD	Sex			Age		
	Male	Female	NA	Children	Adult	NA
normal	4 (36.4%)	6 (54.5%)	1 (9.1%)	11 (100.0%)	0 (0.0%)	0 (0.0%)
VSD	25 (49.0%)	24 (47.0%)	2 (4.0%)	43 (84.3%)	7 (13.7%)	1 (2.0%)
ASD	5 (31.3%)	8 (50%)	3 (18.7%)	11 (68.8%)	2 (12.5%)	3 (18.7%)
AVSD	17 (41.5%)	24 (58.6%)	0 (0.0%)	33 (80.5%)	8 (19.5%)	0 (0.0%)
TGA	33 (61.1%)	20 (37.0%)	1 (1.9%)	51 (94.5%)	3 (5.5%)	0 (0%)
Isomerism	10 (34.5%)	18 (62.1%)	1 (3.4%)	26 (89.7%)	1 (3.4%)	2 (6.9%)
Ebstein	7 (30.4%)	16 (69.6%)	0 (0.0%)	21 (91.3%)	2 (8.7%)	0 (0.0%)
Fallot	18 (52.9%)	16 (47.1%)	0 (0.0%)	30 (88.2%)	4 (11.8%)	0 (0.0%)
TOTAL	119 (45.9%)	132 (51.0%)	8 (3.1%)	226 (87.3%)	27 (10.4%)	6 (2.3%)

VSD, ventricular septal defect; ASD, atrial septal defect; AVSD, atrioventricular septal defect; TGA, transposition of the great arteries; NA, not available.

**Table 4:** Percentage of hearts presenting LVNC according to Chin, Jenni, and Petersen (apex).

	Chin			Jenni			Petersen		
	% (*n*)	OR * (95% CI)	*p*-value	% (*n*)	OR * (95% CI)	*p*-Value	% (*n*)	OR * (95% CI)	*p*-Value
normal	0.0% (0/11)	-	-	0.0% (0/11)	-	-	0.0% (0/11)	-	-
VSD	54.2% (26/48)	27.09 (1.51–486.10)	0.0013	35.4% (17/48)	12.78 (0.71–230.3)	0.0241	12.5% (6/48)	3.52 (0.18–67.21)	0.5820
ASD	8.3% (1/12)	3.00 (0.11–81.68)	1.0000	8.3% (1/12)	3.00 (0.11–81.68)	1.0000	8.3% (1/12)	3.00 (0.11–81.68)	1.0000
AVSD	2.9% (1/34)	1.03 (0.04–27.12)	1.0000	2.9% (1/34)	1.03 (0.04–27.12)	1.000	0.0% (0/34)	0.33 (0.01–17.79)	1.0000
TGA	22.6% (12/53)	6.93 (0.38–126.1)	0.1064	17.0% (9/53)	4.91 (0.27–90.81)	0.3382	5.7% (3/53)	1.59 (0.08–33.07)	1.0000
Isomerism	7.1% (2/28)	2.17 (0.96–48.89)	1.0000	7.1% (2/28)	2.17 (0.096–48.89)	1.000	7.1% (2/28)	2.17 (0.096–48.89)	1.0000
Ebstein	28.6% (6/21)	9.65 (0.49–189.20)	0.0711	9.5% (2/21)	2.95 (0.13–67.00)	0.5343	0.0% (0/21)	0.53 (0.01–28.79)	1.0000
Fallot	5.9% (2/34)	1.77 (0.08–39.70)	1.0000	2.9% (1/34)	1.030 (0.04–27.12)	1.0000	2.9% (1/34)	1.030 (0.04–27.12)	1.0000

* Odds ratio (OR) versus normal hearts.

**Table 5:** Rank of LVNC prevalence among congenital heart diseases according to Chin, Jenni, and Petersen (apex).

	Chin	Jenni	Petersen
1	VSD	VSD	VSD
2	Ebstein	TGA	ASD
3	TGA	Ebstein	Isomerism
4	ASD	ASD	TGA
5	Isomerism	Isomerism	Fallot
6	Fallot	AVSD/Fallot	AVSD/Ebstein
7	AVSD	-	-

**Table 6:** Percentage of hearts presenting LVNC in TGA according to Chin, Jenni, and Petersen (apex).

	Chin			Jenni			Petersen		
CHD	% (*n*)	OR * (95% CI)	*p*-Value	% (*n*)	OR * (95% CI)	*p*-Value	% (*n*)	OR * (95% CI)	*p*-Value
isolated TGA	22.2% (4/18)	-	-	11.1% (2/18)	-	-	5.6% (1/18)	-	-
TGA + ASD	6.7% (1/15)	0.25 (0.02–2.53)	0.3457	6.7% (1/15)	0.57 (0.05–7.0)	1.000	0.0% (0/15)	0.38 (0.01–9.94)	1.0000
TGA + ASD + VSD	57.1% (4/7)	4.67 (0.72–30.12)	0.1563	57.1% (4/7)	10.67 (1.30–86.98)	0.0324	28.6% (2/7)	6.8 (0.51–91.55)	0.1796
TGA + VSD	23.1% (3/13)	1.05 (0.19–5.77)	1.0000	15.4% (2/13)	1.46 (0.18–11.94)	1.0000	0.0% (0/13)	0.43 (0.02–11.47)	1.0000

* Odds ratio (OR) versus isolated TGA.

**Table 7:** Percentage of hearts presenting LVNC in isomerism according to Chin, Jenni, and Petersen (apex).

	Chin			Jenni			Petersen		
CHD	% (*n*)	OR * (95% CI)	*p*-Value	% (*n*)	OR * (95% CI)	*p*-Value	% (*n*)	OR * (95% CI)	*p*-Value
isolated isomerism	0.0% (0/3)	-	-	0.0% (0/3)	-	-	0.0% (0/3)	-	-
isomerism + ASD	0.0% (0/4)	0.78 (0.01–49.95)	1.0000	0.0% (0/4)	0.78 (0.01–49.95)	1.0000	0.0% (0/4)	0.78 (0.01–49.95)	1.0000
isomerism + ASD + VSD	20.0% (1/5)	2.33 (0.70–76.73)	1.0000	20.0% (1/5)	2.33 (0.70–76.73)	1.0000	20.0% (1/5)	2.33 (0.70–76.73)	1.0000
isomerism + AVSD	0.0% (0/8)	0.41 (0.01–25.19)	1.0000	0.0% (0/8)	0.41 (0.01–25.19)	1.000	0.0% (0/8)	0.41 (0.01–25.19)	1.0000
other combinations	11.1% (1/9)	1.23 (0.04–38.33)	1.0000	11.1% (1/9)	1.23 (0.04–38.33)	1.0000	11.1% (1/9)	1.23 (0.04–38.33)	1.0000

* Odds ratio (OR) versus isolated isomerism.

**Table 8:** Percentage of hearts presenting LVNC according to Chin, Jenni, and Petersen when stratified by age in years (y) at the apex.

CHD	Chin		Jenni		Petersen	
	Children (≤18 y)	Adults (>18 y)	Children (≤18 y)	Adults (>18 y)	Children (≤18 y)	Adults (>18 y)
VSD	57.1% (24/42)	40% (2/5)	40.5% (17/42)	0.0% (0/5)	14.3% (6/42)	0.0% (0/5)
ASD	11.1% (1/9)	0.0% (0/1)	11.1% (1/9)	0.0% (0/1)	11.1% (1/9)	0.0% (0/1)
AVSD	3.6% (1/28)	0.0% (0/6)	3.6% (1/28)	0.0% (0/6)	0.0% (0/28)	0.0% (0/6)
TGA	22% (11/50)	33.3% (1/3)	18% (9/50)	0.0% (0/3)	6% (3/50)	0.0% (0/3)
Isomerism	8% (2/25)	0.0% (0/2)	8% (2/25)	0.0% (0/2)	8% (2/25)	0.0% (0/2)
Ebstein	31.6% (6/19)	0.0% (0/2)	10.5% (2/19)	0.0% (0/2)	0.0% (0/19)	0.0% (0/2)
Fallot	6.7% (2/30)	0.0% (0/4)	3.3% (1/30)	0.0% (0/4)	3.3% (1/30)	0.0% (0/4)
TOTAL	23.2% (47/203)	13.0% (3/23)	16.3% (33/203)	0.0% (0/23)	6.4% (13/203)	0.0% (0/23)

Not applicable for normal hearts.

**Table 9:** Percentage of hearts presenting LVNC according to Chin, Jenni, and Petersen when stratified by sex (apex).

CHD	Chin				Jenni				Petersen			
	Male	Female	OR (95% CI)	*p*-Value	Male	Female	OR (95% CI)	*p*-Value	Male	Female	OR (95% CI)	*p*-Value
VSD	41.7% (10/24)	68.2% (15/22)	3.00 (0.89–10.06)	0.0852	25.0% (6/24)	45.5% (10/22)	2.50 (0.72–8.71)	0.2167	0.0% (0/24)	22.7% (5/22)	15.40 (0.80–297.2)	0.0192
ASD	25.0% (1/4)	0.0% (0/6)	0.17 (0.01–5.68)	0.4000	25.0% (1/4)	0.0% (0/6)	0.18 (0.01–5.68)	0.4000	25.0% (1/4)	0.0% (0/6)	0.18 (0.01–5.68)	0.4000
AVSD	7.1% (1/14)	0.0% (0/20)	0.22 (0.01–5.80)	0.4118	7.1% (1/14)	0.0% (0/20)	0.22 (0.01–5.80)	0.4118	0.0% (0/14)	0.0% (0/20)	0.70 (0.01–37.79)	1.0000
TGA	18.8% (6/32)	25.0% (5/20)	1.44 (0.38–5.55)	0.7300	15.6% (5/32)	15% (3/20)	0.95 (0.20–4.51)	1.0000	3.1% (1/32)	10% (2/20)	3.44 (0.29–40.74)	0.5511
Isomerism	10.0% (1/10)	5.9% (1/17)	0.56 (0.03–10.12)	1.0000	10.0% (1/10)	5.9% (1/17)	0.56 (0.03–10.12)	1.0000	10.0% (1/10)	5.9% (1/17)	0.56 (0.03–10.12)	1.0000
Ebstein	14.3% (1/7)	35.7% (5/14)	3.33 (0.31–36.13)	0.6126	0.0% (0/7)	14.3% (2/14)	3.00 (0.13–71.37)	0.5333	0.0% (0/7)	0.0% (0/14)	0.52 (0.01–28.78)	1.0000
Fallot	11.1% (2/18)	0.0% (0/16)	0.20 (0.01–4.50)	0.8889	15.6% (1/18)	0.0% (0/16)	0.35 (0.01–9.31)	1.0000	15.6% (1/18)	0.0% (0/16)	0.35 (0.01–9.31)	1.0000
TOTAL	20.2% (22/109)	22.6% (26/115)	1.06 (0.90–1.24)	0.7551	13.8% (15/109)	13.9% (16/115)	1.01 (0.47–2.16)	1.0000	3.7% (4/109)	7.0% (8/115)	1.963 (0.57–6.71)	0.3766

Not applicable for normal hearts.
